# Radiotherapy plus EGFR TKIs in non‐small cell lung cancer patients with brain metastases: an update meta‐analysis

**DOI:** 10.1002/cam4.673

**Published:** 2016-03-14

**Authors:** Tao Jiang, Weijie Min, Yanan Li, Zhijian Yue, Chunyan Wu, Caicun Zhou

**Affiliations:** ^1^ Department of Medical OncologyShanghai Pulmonary HospitalTongji University School of Medicine507 Zhengmin RoadYangpu DistrictShanghai200433China; ^2^ Department of NeurosurgeryChanghai HospitalSecond Military Medical University168 Changhai RoadYangpu DistrictShanghai200433China; ^3^ Department of PathologyShanghai Pulmonary HospitalTongji University School of Medicine507 Zhengmin RoadYangpu DistrictShanghai200433China

**Keywords:** Brain metastases, EGFR TKI, meta‐analysis, non‐small cell lung cancer, radiotherapy

## Abstract

Brain metastasis (BM) is the common complication of non‐small cell lung cancer (NSCLC) with a poor prognosis and dismal survival rate. This update meta‐analysis aimed to derive a more precise estimation of radiotherapy plus epidermal growth factor receptor (EGFR) tyrosine kinase inhibitors (TKIs) in NSCLC patients with BM. PubMed, EMBASE, Web of Science, Google Scholar, and Cochrane Library were searched to identify any relevant publications. After screening the literature and undertaking quality assessment and data extraction, the meta‐analysis was performed using STATA Version 12.0. In total, 15 studies involving 1552 participants were included. The results indicated that radiotherapy plus EGFR TKIs was more effective at improving response rate and disease control rate (DCR) (risk ratio (RR) = 1.48, 95% confidence interval [CI]: 1.12–1.96, *P *=* *0.005; RR = 1.29, 95% CI: 1.02–1.60, *P *=* *0.035; respectively) than radiotherapy alone or plus chemotherapy. Moreover, radiotherapy plus EGFR TKIs significantly prolonged the time to central nervous system progression (CNS‐TTP) (HR = 0.56, 95% CI [0.33, 0.80]; *P *=* *0.000) and median overall survival (OS) (HR = 0.58, 95% CI [0.42, 0.74]; *P *=* *0.000) but significantly increased adverse events (any grade) (RR = 1.25, 95% CI [1.01, 1.57]; *P *=* *0.009), especially rash and dry skin. These results suggested that radiotherapy plus EGFR TKIs produced superior response rate and DCR and markedly prolonged the CNS‐TTP and OS of NSCLC patients with BM. However, combined groups had the higher rate of incidence of overall adverse effects, especially rash and dry skin.

## Introduction

Lung cancer is characterized by a high incidence of central nervous system (CNS) metastases, with approximately 40% of patients developing brain metastases (BM) in the course of their disease 1, 2, 3, 4. In particular, it has also been estimated that 25–30% of newly diagnosed non‐small cell lung cancer (NSCLC) patients, who account for a large percentage of lung cancer cases, would suffer from BM 5. Patients with NSCLC who develop BM often have poor prognoses. The median overall survival (OS) time was 7 months, and 1‐year survival rate was 20% in one large series 6. Other studies reported that the OS for NSCLC patients with BM is less than 3–6 months when left untreated. Current treatment options include surgery, whole brain radiation therapy (WBRT), stereotactic radiosurgery (SRS) alone or in combination with other strategies such as chemotherapy and targeted therapy.

Radiotherapy including WBRT and SRS play a critical role in the current treatment of NSCLC patients with BM. They are the cornerstone treatment for patients with BM with the choice of radiation technique dependent on the prognosis of the patients and tumor characteristics such as number, size, and site of lesions 7, 8. Traditionally, patients with multiple BM are treated with WBRT to decrease and delay symptoms of increased intracranial pressure as well as to prevent neurological sequelae. In patients with limited number of BM, usually up to three to four lesions, local treatment (SRS or surgery) should be strongly considered.

Epidermal growth factor receptor (EGFR) tyrosine kinase inhibitors (TKIs) are the standard therapy for advanced NSCLC patients with EGFR‐activated mutations based on some famous phase III trials 9, 10, 11. Recent preclinical studies demonstrated that EGFR TKIs might have synergistic effect in combination with radiotherapy on tumor control 12, 13. Erlotinib has been shown to cross the blood–brain barrier (BBB) and may be used to improve the effects of WBRT 14. Some studies indicated that radiotherapy plus EGFR TKIs is more suitable to treat multiple brain lesions of metastatic NSCLC than radiotherapy alone or radiotherapy plus chemotherapy, and showed favorable efficacy and safety 15, 16, 17. However, other studies reported that radiotherapy plus EGFR TKIs showed no advantage in neurological progression‐free survival (PFS) or OS 18. What is worse, some studies suggested that radiotherapy plus EGFR TKIs would lead to poorer survival and much more adverse effects (AEs) than control groups 19.

Whether radiotherapy plus EGFR TKIs has superior efficacy and safety than radiotherapy alone or radiotherapy plus chemotherapy remains controversial. Although there has been a meta‐analysis on this topic, only eight publications were included in that meta‐analysis 20. There have been more than seven papers published since this meta‐analysis was conducted. Moreover, it did not assess some common AEs such as dry skin, anemia, and anorexia in two groups. For these reasons, we performed this update meta‐analysis to derive a more precise estimation of evaluation of radiotherapy plus EGFR TKIs in NSCLC patients with BM.

## Materials and Methods

### Search strategy

PubMed, EMBASE, Web of Science, Google Scholar, and Cochrane Library were searched to identify relevant trials up to June 2015 without language restrictions. Searches were limited to human studies. The main keywords used for the online search were lung neoplasms, lung tumor, lung cancer, brain metastasis, brain neoplasms, radiotherapy, and tyrosine kinase inhibitors.

### Inclusion criteria

All articles which met the following criteria were eligible: (1) prospective or retrospective studies; (2) patients had histologically or cytologically confirmed NSCLC and had been diagnosed with multiple BM using CT or MRI; (3) the trials compared radiotherapy (WBRT/SRS/3D‐CRT alone or in combination) plus EGFR TKIs with conventional chemotherapy plus radiotherapy or radiotherapy or TKIs alone and patients who received prior EGFR TKIs would be excluded; (4) trials did not include patients with chemotherapy contraindications or serious vital organ dysfunction; (5) the analyses included response rate, median survival time (MST), time to CNS progression (CNS‐TTP), PFS, OS, AEs (Grade ≥3), or hematological toxicity (Grade ≥3); (6) response rate was determined using the Response Evaluation Criteria in Solid Tumors or WHO evaluation criteria on solid tumors. Complete remission was defined as tumor completely disappearing for at least 4 weeks without any new lesions. Partial response was defined as more than 50% tumor regression for at least for 4 weeks without new lesions. Progressive disease was defined as an increase in the sum of the longest diameters (LD) of the target lesions by 25% or higher, using as reference the smallest sum LD recorded since treatment started or the appearance of one or more new lesions. Stabilized disease was defined as ≤50% tumor regression or an increase ≤25%. (7) Toxicity was evaluated according to the National Cancer Institute Common Terminology Criteria for Adverse Events.

### Study selection

The eligibility assessment was first performed by screening titles and abstracts and subsequently reviewing the full text of articles. According to the inclusion criteria, two reviewers performed the selection of all studies independently. The third reviewer resolved the disagreement on whether an article should be included.

### Data extraction

Two reviewers independently conducted data extraction in accordance with the Preferred Reporting Items for Systematic Reviews and Meta‐analyses statement (PRISMA). The following items were collected from each study: first author's name, published year, type of study, country of origin study, percentage of female, performance status, number of patients, average ages, interventions, outcomes and toxicity or AEs.

### Quality assessment

Two reviewers independently assessed the risk of bias of the included studies according to The Cochrane Handbook for Systematic Reviews (Version 5.1.0), based on the following criteria: (1) Random sequence generation; (2) Allocation concealment; (3) Blinding of participants and personnel; (4) Blinding of outcome assessment; (5) Incomplete outcome data; (6) Selective reporting; (7) Other bias. Each trial for bias based on the criteria listed above was marked as “low risk,” “high risk,” or “unclear risk.” Trials were judged as low risk of bias (i.e. A rating) when all criteria were assessed as low risk; trials were judged as moderate risk of bias (i.e. B rating) or high risk of bias (i.e. C rating) when one or more criteria were assessed as unclear risk or high risk, respectively.

### Statistical analysis

Statistical analyses were performed using STATA Version 12.0 (Stata Corporation LP, College Station, TX). Chi‐squared and *I* ^2^ tests were used to test the heterogeneity of different studies. For time‐to‐event data, the HRs with 95% CIs were directly extracted from the research article or calculated using previously published methods proposed by Tierney et al. 21. The *I* ^2^ test was used to test for statistical heterogeneity and the *I* ^2^ statistic was used to assess the extent of variability attributable to statistical heterogeneity across trials. *P* > 0.1 for the *I* ^2^ test and *I* ^2^ < 25% were interpreted as signifying low‐level heterogeneity. When there was no statistically significant heterogeneity, a pooled effect was calculated with a fixed‐effects model; otherwise, a random‐effects model was used. Response rate, severe hematological toxicity, and adverse events were analyzed using dichotomous variables. PFS and OS were calculated using effect variables.

## Results

### Selection of studies

**Figure 1:**
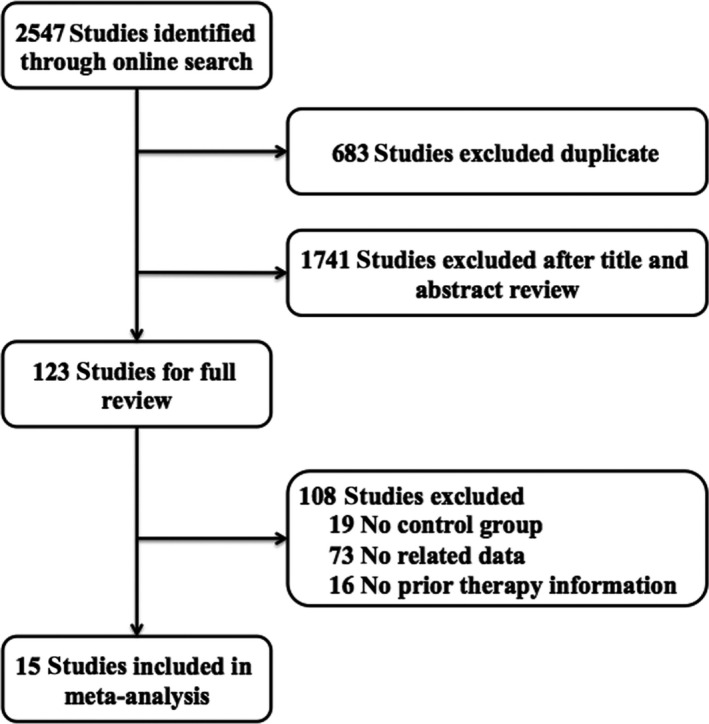
Flow chart of studies included in the meta‐analysis.

Totally, we identified 2547 studies that met our selection criteria after searching the relevant databases; 683 of these studies were excluded due to duplications. By verifying related terms in the titles and abstracts, we excluded 1741 irrelevant articles, and another 108 articles were excluded after the full text was read. Finally, 15 studies were selected for the present meta‐analysis 15, 16, 17, 18, 19, 22, 23, 24, 25, 26, 27, 28, 29, 30, 31. A flowchart depicting the study selection is shown in Figure 1.

### Characteristics of included studies

There were a total of 1552 NSCLC patients with BM originating from NSCLC in the included studies, with 672 patients having received radiotherapy plus EGFR TKIs. The characteristics of the included trials are summarized in Table 1. Of the 15 studies, four studies were prospective studies including one phase III clinical trial and three phase II trials 17, 18, 19, 22. The analyzed interventions were WBRT/SRS plus EGFR TKIs and WBRT/SRS alone or WBRT/SRS plus chemotherapy, except in the case of Sperduto et al. 2013, which compared the combination treatment WBRT, SRS, and erlotinib with WBRT+SRS treatment. Especially, one study analyzed the 3D‐CRT plus TKI and 3D‐CRT plus VM‐26 (teniposide). Among all of the included studies, EGFR TKIs included gefitinib and erlotinib. Conventional chemotherapy drugs included placebo, temozolomide (TMZ), VMP, pemetrexed, gemcitabine, platinum, and other chemotherapy agents. Ten studies reported sufficient data for RR and DCR. Three studies reported CNS‐TTP and eight studies reported OS results. Eight studies reported AE data. Of note, one study recorded the Kaplan–Meier curve of PFS and OS. To avoid selection bias, we did not extract HRs with 95% CIs from the published figures.

**Table 1:** Characteristics of the included studies

Author	Year	Country	Trial phase	Patient no. (T/C)	Median ages (T/C, years)	Female (T/C, %)	EGFR mutation (%)	Treatment group	Treatment pattern	Control group	Outcomes	Study quality
Pesce	2012	Switzerland	II	16/43	57/63	44.0/37.0	NA	WBRT+gefitinib	Concurrent	WBRT+TMZ	mOS	B
Fu	2012	China	NA	38/123	NA	NA	NA	WBRT/SRS+gefitinib	Concurrent	WBRT/SRS	ORR	C
Zeng	2012	China	NA	45/45	56/52	57.8/53.3	NA	WBRT+gefitinib	Concurrent	Gefitinib	RR, DCR, mTTP, mPFS, mOS	B
Wu	2012	China	NA	35/18	NA	NA	NA	WBRT+gefitinib	Concurrent	WBRT	RR, DCR, mOS	B
Cai	2013	China	NA	65/92	NA	38.5/31.5	27.4	WBRT+gefitinib/erlotinib	Concurrent	WBRT	RR, DCR, mPFS, mOS	B
Sperduto	2013	Multicenter	III	41/44	61/64	NA	NA	WBRT+SRS+erlotinib	Concurrent	WBRT/SRS	CNS‐TTP, mOS	B
Zhuang	2013	China	II	23/31	60/63	57/58	20.4	WBRT+erlotinib	Concurrent	WBRT	ORR, CNS‐TTP, PFS, mOS	B
Liu	2013	China	NA	52/52	54/51	44.2/48.1	NA	WBRT+SRS+gefitinib/erlotinib	Concurrent	WBRT+SRS	RR, DCR, PFS	C
Zhou	2013	China	NA	36/22	NA	58.3/50.0	NA	WBRT+gefitinib/erlotinib	Concurrent	WBRT+chemotherapy	RR, DCR, mOS	C
Fan	2013	China	NA	75/111	57/57	42.7/27.0	NA	WBRT/SRS+TKI	Sequential	WBRT/SRS+chemotherapy	mOS	B
Lee	2014	Britain	II	40/40	61/62	62.5/47.5	2.9	WBRT+erlotinib	Concurrent	WBRT+placebo	CNS‐TTP, mOS	B
Cai	2014	China	NA	104/178	65/65	40.4/33.7	19.50%	WBRT/SRS+TKI	Sequential	WBRT/SRS	CNS‐TTP, mOS	B
Wang	2015	China	NA	37/36	61/62	32.4/36.1	12.30%	3D‐CRT+gefitinib	Concurrent	3D‐CRT+chemotherapy	ORR, mOS	B
Liu	2015	China	NA	35/15	46.3/47.5	51.4/53.3	NA	WBRT+gefitinib	Concurrent	WBRT+chemotherapy	RR, DCR, mOS	C
Chen	2015	China	NA	30/30	64.5/64.3	46.7/50.0	NA	WBRT+gefitinib	Concurrent	WBRT+chemotherapy	RR, DCR, PFS, mOS	B

T/C, treatment/control; ECOG PS, eastern cooperative oncology group performance status; NA, not applicable; RR, response rate; DCR, disease control rate; TTP, time to progression; PFS, progression‐free survival; OS, overall survival; WBRT, whole brain radiotherapy; SRS, stereotactic radiosurgery; CNS, central nervous system; 3D‐CRT, three‐dimensional conformal radiotherapy; TMZ, temozolomide.

### Methodological quality

In accordance with the recommendations of the Cochrane Handbook for Systematic Reviews, we evaluated the eligible studies using the aspects mentioned above. Four studies 16, 18, 19, 22 mentioned the use of random allocation, but only two of them discussed the methods 19, 22. One study performed or reported their allocation concealment and blinding methods 17. None of the trials reported follow‐up information. All of the articles applied the intent‐to‐treat analysis. Eleven of the 15 eligible studies received B quality scores and four received C quality scores, as shown in Table 1.

### Response rate and disease control rate

**Figure 2:**
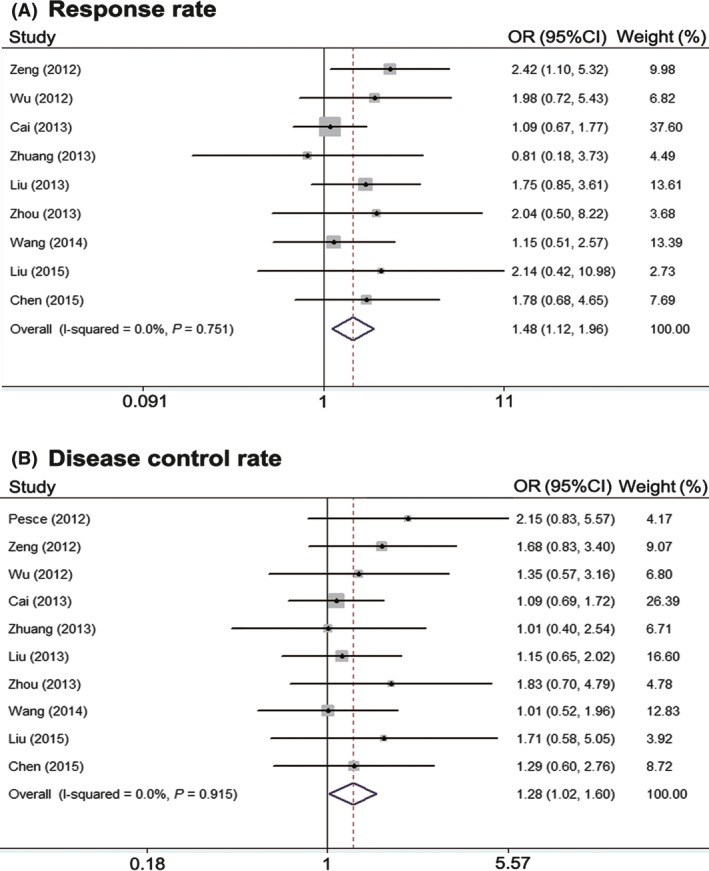
Meta‐analysis of response rate (A) and disease control rate (B).

Ten of the included studies reported the RR and DCR of treatment using radiotherapy plus TKIs and radiotherapy alone or radiotherapy plus chemotherapy 16, 17, 22, 23, 24, 27, 28, 29, 30, 31. RR ranged from 13.0% to 77.1% in the radiotherapy plus TKI groups and 13.3–70.7% in control groups. DCR ranged from 52.2% to 97.1% in the radiotherapy plus TKI groups and 36.4–89.1% in control groups. There was no heterogeneity among the 10 studies in both RR and DCR (*P *=* *0.751, *I* ^2^ = 0.0%; *P *=* *0.915, *I* ^2^ = 0.0%; respectively), and as a result, the fixed effect model was used for the meta‐analysis. The pooled results indicate that radiotherapy plus TKIs resulted in superior RR and DCR when compared with control groups (RR = 1.48, 95% CI: 1.12–1.96, *P *=* *0.005; RR = 1.29, 95% CI: 1.02–1.60, *P *=* *0.035; respectively) (Fig. 2A and B).

### Survival

**Figure 3:**
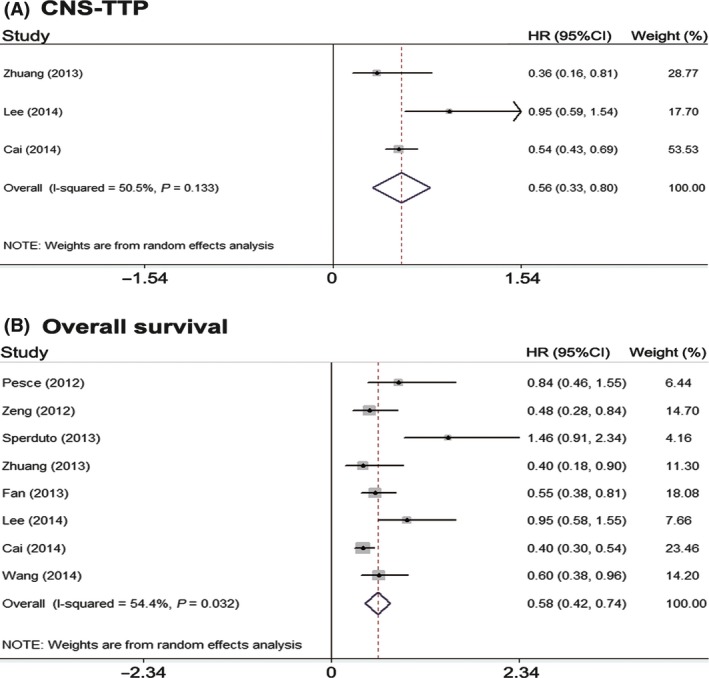
Meta‐analysis of time to central nervous system progression (A) and median overall survival (B).

Three studies reported CNS‐TTP 15, 17, 18. The studies were heterogeneous (*P *=* *0.133, *I* ^2^ = 50.5%). Analysis using a random effect model suggests that in NSCLC patients diagnosed with BM, there was the significantly better CNS‐TTP in radiotherapy plus TKIs groups than in radiotherapy alone or radiotherapy plus chemotherapy (HR = 0.56, 95% CI: 0.33–0.80; *P *=* *0.000) (Fig. 3A). Eight studies reported OS results and there was a significant heterogeneity between them (*P *=* *0.032, *I* ^2^ = 54.4%) 15, 16, 17, 18, 19, 22, 23, 25. Accordingly, a random effect model was used for the meta‐analysis of OS. The results suggest that combining radiotherapy with TKIs could prolong OS (HR = 0.58, 95% CI: 0.42–0.74; *P *=* *0.000) (Fig. 3B). In a subgroup analysis, we compared the different results between concurrent and sequential treatment. Only two studies reported the HR of CNS‐TTP and OS of sequential treatment 15, 25. After excluding them, we found that concurrent radiotherapy plus EGFR TKIs could also significantly prolong the CNS‐TTP (HR = 0.55, 95% CI: 0.28–0.82; *P *=* *0.000) and OS (HR = 0.63, 95% CI: 0.45–0.81; *P *=* *0.000) of NSCLC patients with BM (Fig. S1).

### Adverse effects

Eight enrolled studies had analyzed the treatment‐related toxicity and AEs. A fixed‐effects model was used for the overall AE analysis of these studies based on the heterogeneity values (*P *=* *0.213, *I* ^2^ **= 12.9%). The results indicate that the incidence of overall AEs was higher in the group treated using radiotherapy plus TKIs (RR = 1.25, 95% CI: 1.01–1.57; *P *=* *0.009). The most common adverse events of TKIs are rash, fatigue, nausea/vomiting, and diarrhea, which are largely mild and fairly tolerable, and pneumonitis rarely occurs. Thus, we performed a subgroup analysis for these AEs as showed in Table 2. Regarding nausea/vomiting, diarrhea, pneumonitis, and other AEs, no difference was observed. However, rashes (RR = 4.97, 95% CI: 2.68–9.21; *P *=* *0.000) and dry skin (RR = 8.44, 95% CI: 1.48–48.28; *P *=* *0.000) were significantly more common in the radiotherapy plus TKI group.

**Table 2:** Meta‐analysis of the reported common adverse effects in the included studies

Adverse effect	Number of studies	Test of association			Test of heterogeneity		
		RR	95% CI	*P*	*Q*	*I* ^2^%	*P*
Fatigue	5	0.762	0.418–1.388	0.374	1.92	0.0	0.751
Dyspnea	2	2.287	0.193–27.116	0.512	2.66	62.4	0.103
Rash	8	4.971	2.683–9.208	0.000	8.33	15.9	0.305
Anorexia	2	1.634	0.677–3.944	0.275	1.22	18.0	0.270
Diarrhea	8	1.506	0.938–2.417	0.090	5.00	0.0	0.660
Headache	4	1.070	0.657–1.743	0.786	2.68	0.0	0.443
Anemia	3	1.324	0.616–2.846	0.472	2.59	22.8	0.274
Constipation	2	1.799	0.652–4.964	0.256	0.11	0.0	0.739
Dry skin	2	8.438	1.475–48.278	0.017	0.50	0.0	0.477
Nausea	8	0.979	0.682–1.407	0.910	9.54	26.6	0.216
Pneumonitis	2	1.108	0.375–3.273	0.852	0.64	0.0	0.425
Total	8	1.247	1.012–1.572	0.009	6.99	12.9	0.213

RR, risk rate; CI, confidence interval.

## Discussion

Currently, local radiotherapy remains the standard therapy of NSCLC patients with BM. Several studies have verified that WBRT and/or SRS could palliate the neurological symptoms associated with BM. However, the prognosis for NSCLC patients with BM is poor. Overall, median survival of BM patients is about 2.4–4.8 months after WBRT 32, 33. Recent studies of radiotherapy in combination with conventional chemotherapeutic drugs, such as platinum, paclitaxel, and TMZ, suggest no significant improvement in OS compared with radiotherapy alone owing to their low capacity of penetrating the BBB 34, 35, 36, 37. As the small molecular drugs, TKIs such as gefitinib and erlotinib have the possibility of crossing the BBB. Previous studies demonstrated that gefitinib and erlotinib showed good permeability through the BBB 38, 39, 40. After penetrating into the BBB, TKIs can compete with adenosine triphosphate and provide sufficient radiosensitizing and therapeutic level in the brain to exert their anti‐cancer efficacy 13, 14, 41. Hence radiotherapy plus EGFR TKIs seems to have the promising antitumor effect.

In our study, we demonstrated that radiotherapy plus EGFR TKIs produced superior RR and DCR and markedly prolonged the CNS‐TTP and OS of NSCLC patients with BM. However, combined group increased the incidence of overall AEs, especially rash and dry skin, which may be attributed to TKI therapy. The possible reason about these results may include that the patients in most of the included studies come from East Asia. As is known, East Asian patients with NSCLC have a higher EGFR mutation rate than patients from other ethnicities. In East Asian patients, Matsumoto et al. and Gow et al. have found EGFR mutations in 63% and 44% of BM, respectively 42, 43. This prevalence is similar to that reported in primary tumors of the same population, varying from 30% to 50% 44, 45. In Caucasian cohorts, with a low overall prevalence of EGFR mutations, activating mutations were found in 0–2% of BM 46, 47. In addition, the EGFR mutation rate was 64% and 31% in patients with and without BM, respectively, suggesting that BM would be more frequent in patients with tumors bearing EGFR mutations. However, another two studies with small sample sizes have suggested a discordant EGFR mutation rate between primary and brain metastatic tumors between 0% and 32% 48, 49. Therefore, in future, we need to explore how to precisely select patients with the concordant EGFR mutation between primary lesions and BM. For patients with the discordant EGFR mutation between primary lesions and BM, we need to explore the possible molecular mechanism during the process of BM with the help of advanced techniques such as next generation sequencing.

Considering the effect of WBRT on BBB, which would increase the cerebral concentration of TKIs, we performed subgroup analysis to compare the different results between concurrent and sequential treatment. Our result showed that both concurrent and sequential treatment could significantly prolong the CNS‐TTP and OS of NSCLC patients with BM, possibly due to the synergistic effect of EGFR TKI and WBRT 14. WBRT could increase the penetration of EGFR TKI via disturbing BBB and EGFR TKI could increase the anti‐tumor effect of WBRT in BM 5, 15. Further prospective studies are needed to determine the optimal treatment pattern for NSCLC patients with BM.

The treatment of NSCLC has entered the era of precision medicine based on the different driver gene mutations. The survival of patients with advanced NSCLC has been significantly improved. However, NSCLC patients with BM still suffer from dismal prognosis. The major reasons include the unknown molecular mechanism of the BM process and lack of specific therapeutic targets. Fortunately, several recent studies revealed the potential mechanism of lung cancer metastases to brain. Nguyen et al. demonstrated that a distinct WNT/TCF signaling program through LEF1 and HOXB9 enhances the competence of lung adenocarcinoma cells to colonize the bones and the brain. These findings are useful for achieving a deeper understanding of early metastatic events and the development of improved treatments for lung adenocarcinoma patients at risk of BM 50. Another study showed that brain metastatic cells from lung cancer could express high levels of anti‐plasminogen activator serpins, including neuroserpin and serpin B2, to prevent plasmin generation and its metastasis‐suppressive effects, which may become promising targets for NSCLC patients with BM 51. A more recent study illustrated that a disintegrin and metalloproteinase domain 9 (ADAM9) could regulate lung cancer metastasis to the brain by facilitating tissue plasminogen activator‐mediated cleavage of CDCP1, with potential implications to target this network as a strategy to prevent or treat brain metastatic disease 52. We do believe that with the clear molecular mechanisms revealed, the treatment of BM from lung cancer will make great progress in future years.

Our systematic review with meta‐analysis has some limitations that should be acknowledged. First, the number of included studies was relatively small and some of them are retrospective studies. Thus the quality of evidence supporting the findings is low. Second, it is possible that there may be some degree of publication bias in this area of research. We identified several abstracts describing articles that were not further detailed in standard publications; hence, we could not include these articles in the review. Third, the quality of the data was heterogeneous as several pieces of important information such as prior therapy, performance status, number of BM, and health‐related quality of life outcomes were not consistently reported. Notably, all included articles were not randomized controlled studies except one phase III trial 19, so more robust data with high clinical evidence will be included in future analysis. Last but not least, some previous studies have shown that response to radiotherapy was different according to the type of mutation, and particularly some difference may exist according to KRAS amino acid substitution 53. A recent publication has shown that EGFR TKI could increase, in vitro, the radiosensitivity of tumors harboring a KRAS mutation by limiting chromatin condensation induced by EGFR pathway activation 54. Therefore, it would be interesting to add EGFR TKI to radiotherapy that even if EGFR TKI are known to be not effective on patients with KRAS mutations.

In conclusion, the current evidence suggests that radiotherapy plus EGFR TKIs produced superior RR and DCR and markedly prolonged the CNS‐TTP and OS of NSCLC patients with BM. Meanwhile, combined group increased the incidence of overall AEs, especially rash and dry skin. In future, more high‐quality and large‐scale clinical trials are necessary to confirm the efficacy and safety of radiotherapy plus EGFR TKIs and select the most benefit population in NSCLC patients with BM.

## Conflict of interest

The authors have no conflicts of interest to declare.

## Supporting information


**Figure S1.** Meta‐analysis of time to central nervous system progression (A) and median overall survival (B) in concurrent radiotherapy and EGFR TKI group.
