# Supplementary figures and images for: Radiotherapy plus EGFR TKIs in non‐small cell lung cancer patients with brain metastases: an update meta‐analysis
Source: Cancer Med. 2016 Mar 14;5(6):1055–65. doi: 10.1002/cam4.673 (PMC4924363; doi:10.1002/cam4.673)

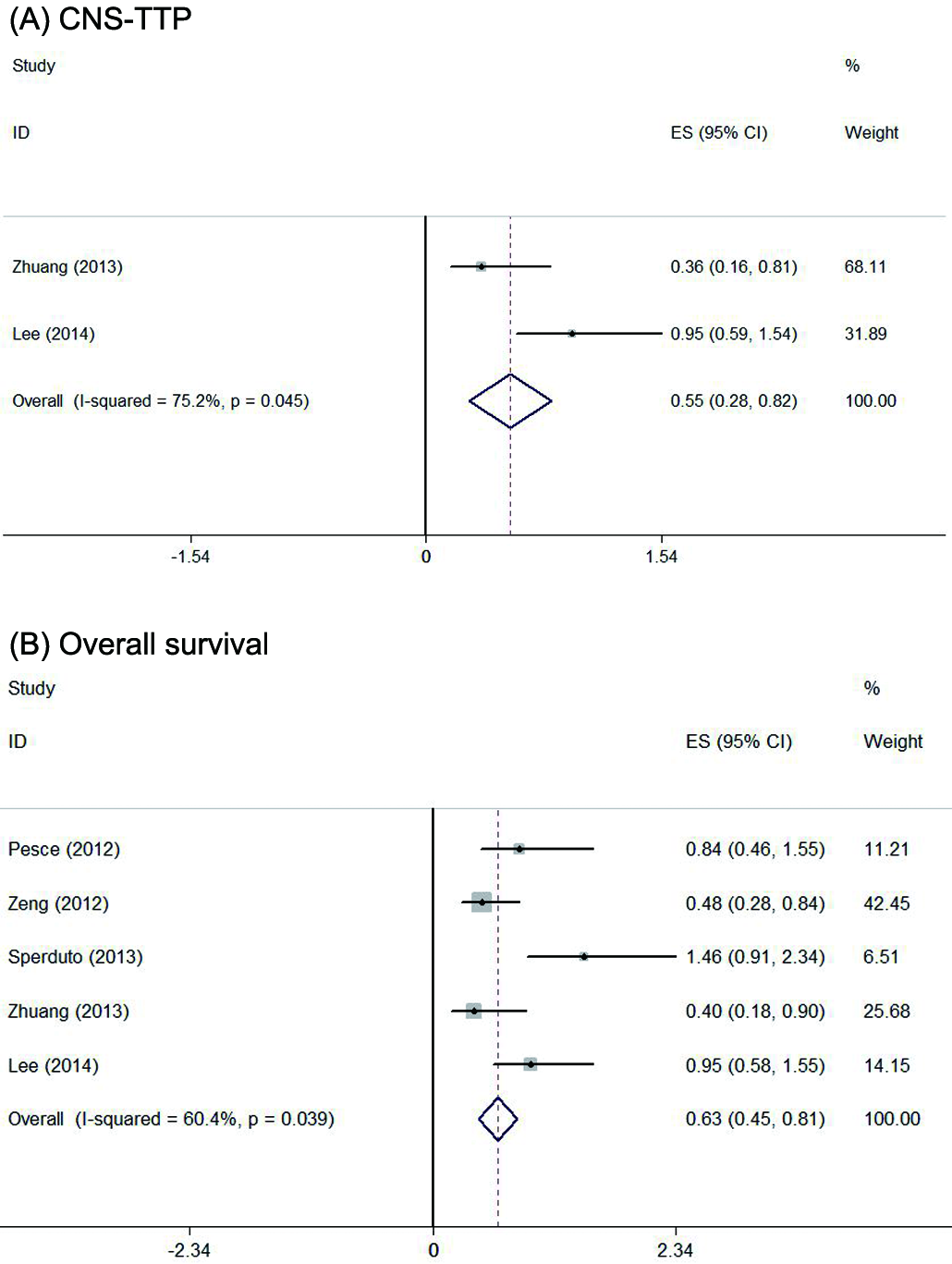

Supplement: Supplementary file 1 — Figure S1. Meta‐analysis of time to central nervous system progression (A) and median overall survival (B) in concurrent radiotherapy and EGFR TKI group. [file CAM4-5-1055-s001.tiff]
